# Non-canonical Targets Mediating the Action of Drugs of Abuse: Cocaine at the Sigma-1 Receptor as an Example

**DOI:** 10.3389/fnins.2019.00761

**Published:** 2019-07-23

**Authors:** Tsung-Ping Su

**Affiliations:** Cellular Pathobiology Section, Integrative Neuroscience Research Branch, Intramural Research Program, National Institute on Drug Abuse, National Institutes of Health, DHHS, Baltimore, MD, United States

**Keywords:** methampetamine, nicotine, opioid, cocaine, intracellular action, sigma-1 receptor, inside-out action, morphine

## Abstract

In addition to acting on traditionally recognized receptors or transporters on the plasma membrane, several drugs of abuse, including amphetamine, methamphetamine, nicotine, opioid, cocaine, ketamine, and cannabinoid, have been shown to exert their effects by acting on additional molecular targets either on the plasma membrane or inside a cell. These targets are usually nascent receptors or proteins that can cause downstream signaling or molecular events, leading to altered physiological outcomes favoring addictive processes. However, those “non-canonical” targets of drugs of abuse, in general, have not been widely recognized in drug abuse research. This perspective diverts attention to those underrecognized targets, in the hope of promoting a more complete understanding of the action of drugs of abuse.

The canonical targets of the action of drugs of abuse are thought to be receptors or transporters on the plasma membrane that, in turn, trigger the downstream signaling to yield physiological responses. While this mode of action of drugs of abuse has been widely recognized and has seemingly dominated the whole field of research, the notion of other non-canonical targets, if any, that mediate the effect of drugs of abuse has not been well recognized. This perspective summarizes certain drugs of abuse that have been shown to mediate their effects through non-canonical targets. The action of cocaine at the sigma-1 receptor (Sig-1R) is given here as an example.

The first example of such a non-canonical target of the action of a drug of abuse came from a study on amphetamine. Sulzer and Rayport (1990) found that in cultured midbrain, dopaminergic neurons amphetamine alkalinizes the intracellular pH gradient that is essential for dopamine uptake into synaptic vesicles. They used isolated adrenal chromaffin granules, which resemble synaptic vesicles, and showed that amphetamine inhibits the acidic gradient in isolated monoaminergic vesicles. A later study supported this result by demonstrating that in dopamine transporter (DAT)-knockout animals the vesicular dopamine-depleting action of amphetamine is preserved (Jones et al., 1998), again suggesting a DAT-independent intracellular action of amphetamine. The second example is methamphetamine. In Asanuma et al. (2000), reported that methamphetamine may exist intracellularly and affect directly the binding of transcription factors AP and cAMP response element-binding protein (CREB) to DNA. Methamphetamine may thus affect the transcriptional regulation of certain genes by working directly in the nucleus (Asanuma et al., 2000). In addition, a recent study on the effect of methamphetamine on the DAT (Sambo et al., 2017) may be interpreted as methamphetamine acting from inside of a neuron on an unknown target to affect the conformation of the DAT.

The third abused drug working in this manner is nicotine. Sallette et al. (2005) reported that the site of action of nicotine in causing an overall upregulation of subunits of nicotinic acetylcholine receptor is inside the cell.

Henderson and Lester (2015) therefore coined the term “inside-out” to describe this non-canonical target of nicotine, which has been the drug of interest in their research for years. Further, Lester’s group experimentally determined, by using specific biosensors of nicotine, that nicotine indeed exists inside a cell and acts as a molecular chaperone to assist in the maturation of nicotine acetylcholine receptors and thus upregulates their subunits (Shivange et al., 2019).

Interestingly, this non-canonical target of action was also reported in 2018 by von Zastrow’s group in the effect of morphine and etorphine. Those two opioid alkaloids were, in part, presumably freely diffused into the inside of a cell (Stoeber et al., 2018). The report discovered that the intracellular *mu* opioid receptor was in fact activated by the intracellular morphine. The intracellularly activated *mu* opioid receptor could be deactivated by the membrane-permeable opioid antagonist naloxone. In addition, the signaling consequence of the internal activation of *mu* opioid receptors by opioid alkaloids is different from that activated by the opioid receptor on the plasma membrane by opioid peptides (Stoeber et al., 2018). This is, perhaps, the first time a non-canonical target of an opioid is described.

Here, a non-canonical target mediating the action of cocaine is presented. Cocaine is well known to increase the level of synaptic dopamine by binding to the DAT on the plasma membrane. However, cocaine has been demonstrated over the past few years to work through a non-canonical target called the sigma-1 receptor (Sig-1R).

The Sig-1R is an intracellular ligand-regulated molecular chaperone that resides mainly at the endoplasmic reticulum (ER)-mitochondria contact referred to as the MAM (Hayashi and Su, 2007). The Sig-1R, upon stimulation by its agonist, can translocate to other parts of the cell to bind and regulate the function of many proteins, including receptors and ion channels (Su et al., 2016). Thus, the Sig-1R plays many diverse roles in cellular functions. Examples of several of the Sig-1R functions are given as follows. At the MAM, the Sig-1R ensures proper Ca^2+^ signaling from the ER into mitochondria by chaperoning IP3 receptor type 3 at the MAM (Hayashi and Su, 2007). The Sig-1R can also increase the dendritic spine maturation by attenuating the activity of caspase 3 that would otherwise inactivate Rac1.GTP (Tsai et al., 2009). The Sig-1R promotes axon extension by binding and transferring myristic acid to p35 to ultimately avoid the hyperphosphorylation of tau that would stun the axon extension (Tsai et al., 2015b). At the nuclear membrane, the Sig-1R binds the nuclear envelope protein emerin to recruit chromatin remodeling molecules to regulate gene transcription (Tsai et al., 2015a).

Interestingly, the electron microscope studies show that the Sig-1R can be present in part on the plasma membrane of dorsal root ganglia (Mavlyutov et al., 2016) but can exist only intracellularly in retinal neurons (Mavlyutov et al., 2015). In retinal neurons, the Sig-1R is seen mainly at the ER but exists in part in the subsurface ER cisternae that are juxtaposed to the plasma membrane (Mavlyutov et al., 2015). Thus, the Sig-1R ligands may target the Sig-1R either on the plasma membrane or inside of a cell, depending on the type of cells or neurons.

In addition to working as a DAT blocker on the plasma membrane, cocaine can bind to the Sig-1R (Sharkey et al., 1988; Hiranita et al., 2011), acts as a Sig-1R as an agonist, and causes the dissociation of Sig-1R from its binding partner, the binding immunoglobulin protein (BiP) (Hayashi and Su, 2007), at the MAM. This action of cocaine leads to the translocation of Sig-1R to other parts of neuron, including the plasma membrane (Kourrich et al., 2013) or nuclear membrane (Tsai et al., 2015a). Cocaine thus causes a decreased excitability of GABAergic medium spiny neurons by increasing the association between the Sig-1R and Kv1.2 at the plasma membrane (Kourrich et al., 2013). At the nuclear membrane, this action of cocaine causes an increased association between Sig-1R and emerin, leading to a chromatin remodeling that attenuates the transcription of monoamine oxidase B (Tsai et al., 2015a). Those two actions of cocaine via Sig-1R provide new insight, respectively, into the understanding of behavioral sensitization (Kourrich et al., 2013) and the increase of synaptic dopamine (Tsai et al., 2015a) caused by cocaine. It has to be mentioned that the cultured systems used in those two studies did not contain dopamine nor did they contain any neural circuitry. Thus, these actions of cocaine are not mediated through DAT on the plasma membrane but are mediated through the non-canonical target the Sig-1R. Since the Sig-1R, as mentioned above, may exist on the plasma membrane (Mavlyutov et al., 2016) or inside a neuron (Mavlyutov et al., 2015), cocaine may target the Sig-1R on the plasma membrane or inside a neuron. The next section explains why cocaine, often used as a conjugated salt in research, may pass the plasma membrane to target the Sig-1R inside of a neuron.

The most popular form of cocaine used in research is cocaine hydrochloride which would not pass the plasma membrane because cocaine is protonated in this salt form. Only the unprotonated (base) form of cocaine may pass the plasma membrane because of its lipophilicity and neutrality. In fact, the two studies mentioned above on dendritic spine formation (Tsai et al., 2009) and axon extension (Tsai et al., 2015b) utilized cocaine hydrochloride. How does cocaine enter a cell when it is used in the form of a hydrochloride salt?

According to the principle of chemistry, cocaine hydrochloride should exist in equilibrium in its base form and its protonated form under the physiological solution with a pH at 7.4. This happens because, given the sufficient ionic strength of the physiological buffer, the conjugated proton of cocaine will be forced to conform to or contribute to the proton concentration dictated by the final pH of 7.4. The relative concentration of these two forms of cocaine under the physiological buffer of a pH of 7.4 can be determined by the Henderson-Hasselbalch equation (see below) with the known pKa of cocaine (Lu et al., 2007). Thus, the actual molar concentration of those two forms of cocaine can be obtained. Further, the fractional occupancy of Sig-1R by cocaine inside of a cell can be calculated, from the concentration of the intracellular cocaine, via the Michaelis–Menton equation (Michaelis et al., 2011) in which the binding affinity (Ki) of cocaine at the Sig-1R can be set at 5.19 μM (Hiranita et al., 2011). Assuming that cocaine hydrochloride is used at 10 μM in a test system, details of the calculation on the concentration of intracellular cocaine and its fractional occupancy at Sig-1R are shown as follows.

p⁢H-p⁢K⁢a=l⁢o⁢g⁢[(B⁢a⁢s⁢e)/(p⁢r⁢o⁢t⁢o⁢n⁢a⁢t⁢e⁢d⁢f⁢o⁢r⁢m)]

(Henderson–Hasselbalch equation) (Eq. 1)

C⁢o⁢c⁢a⁢i⁢n⁢e⁢p⁢K⁢a=8.6

Accordingly, the ratio of cocaine base form vs. protonated form at pH 7.4 per Eq. 1 would be:

7.4-8.6=-1.2=l⁢o⁢g⁢(0.063)=1/15.87

Assuming the concentration of cocaine hydrochloride used in the test is 10 μM, the above ratio would indicate the base form concentration to be at 0.59 μM and the protonated form at 9.41 μM. Thus, the total cocaine concentration inside a cell would be 0.59 μM. Further, since the binding affinity (Ki) of cocaine at Sig-1R is 5.19 μM (Hiranita et al., 2011), the fractional occupancy of cocaine at the Sig-1R inside a cell can be calculated according to the Michaelis–Menton equation (Michaelis et al., 2011) as follows.

0.59/[5.19+0.59]=10.2%

Note: A ten micromolar concentration of cocaine used *in vitro* tests, including those in electrophysiological studies, is not too much since we have shown that a 20 mg/kg i.p. injection of cocaine gives rise to at least 30 μM of cocaine in 30 min in the rat neocortex (Lee et al., 2008).

Thus, cocaine ordinarily tested in the form of a hydrochloride salt is able to diffuse through the plasma membrane in the base form to interact with the Sig-1R inside a cell.

In fact, this notion and calculations shown above, by using cocaine as an example, can be applied to other drugs of abuse when they are used in the form of a conjugated salt. In other words, the concentration of the unprotonated form of a drug that will freely diffuse into the neuron to interact with intracellular targets can be known.

A result is presented below illustrating the DAT-independent action of cocaine in the regulation of axon extension in primary neurons.

The effect of cocaine on the axonal extension in mouse primary cortical neurons was examined. The methods on the primary culture have been previously reported in great detail (Tsai et al., 2015b). Briefly, cocaine hydrochloride was added to the fresh culture medium daily from DIV 7 to DIV 10. Neurons were stained for soma and axons by anti-phosphorylated neurofilament heavy polypeptide. Axon length was examined on DIV 10. Note: (1) Synapses were not formed before DIV 13; (2) No dopamine was added into the culture medium.

Primary neurons were treated with saline, or cocaine at 3 μM or 10 μM. Data were collected from three sets of independent experiments and were combined for statistical analyses. Results show that cocaine chloride at 3 μM (*p* < 0.0001 compared to saline group; *n* = 68 axons for saline, *n* = 94 axons for 3 μM of cocaine; two-tailed *t*-test) and 10 μM (*p* < 0.0004 compared to saline group; *n* = 68 axons for saline, *n* = 102 axons for 10 μM of cocaine; two-tailed *t*-test) significantly increased the axon length (Figure 1).

**FIGURE 1:**
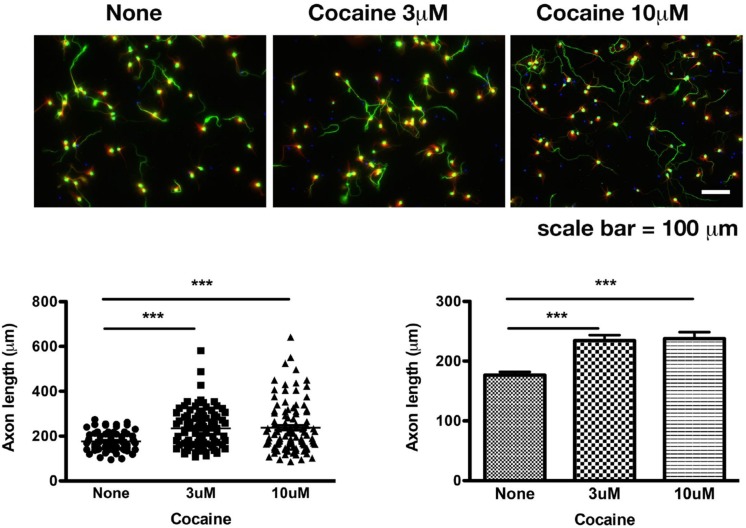
Cocaine increases the length of axons in mouse primary cortical neurons. Cocaine was added into the culture when medium was being refreshed daily from DIV 7 to DIV 10. Data were collected on DIV 10. Note: (1) Dendritic spines were not formed until DIV 14. Therefore, synapses were not present at the time of data collection; (2) There was no dopamine in the medium. Data represent combined results from three sets of independent experiments. Green: axons and soma labeled by pNF-H; Red: dendrites labeled by MAP-2B; Blue: nuclei labeled by DAPI. Total numbers of axons examined were 68, 94, and 102, respectively, for saline, 3 μM cocaine, 10 μM cocaine. ^∗∗∗^*p* < 0.0004 or 0.0001 (two-tailed *t*-test).

As mentioned above, dopamine and synapses were absent in the test system. Therefore, this result of cocaine on axon extension cannot be explained by the effect of cocaine on DAT nor by circuitry connections. This action of cocaine may best be explained by the working of cocaine through a new mechanism. The Sig-1R may mediate this action of cocaine. As mentioned above, the Sig-1R plays a critical role in axonal extension in primary neurons by binding and transferring myristic acid from the ER to the plasma membrane to facilitate the degradation of p35 that would otherwise hamper the axon extension by causing the hyperphosphorylation of tau (Tsai et al., 2015b). Thus, given that cocaine is a Sig-1R agonist (Hayashi and Su, 2007) and that the Sig-1R may exist on the plasma membrane or inside a neuron, cocaine may increase axon extension by targeting the Sig-1R either on the plasma membrane or at the inside of a neuron. From inside a neuron, cocaine may mobilize the Sig-1R from the MAM to plasma membrane to facilitate the myristoylation of p35 (Tsai et al., 2015b). At the plasma membrane, cocaine may directly facilitate the interaction between the Sig-1R and, for example, potassium channel Kv1.4 (Aydar et al., 2002) or other ion channels to achieve its action. At any rate, cocaine may act in part through the non-canonical target of the Sig-1R to achieve a neuroplasticity favoring its addictive processes.

Ketamine may also work through a non-canonical target inside of a neuron (Lester et al., 2015). Although the antidepressant action of ketamine was shown to be mediated through the NMDA receptor on the plasma membrane (Yang et al., 2018), the actual molecular target of ketamine remains unknown (Zanos et al., 2016) and has been speculated to potentially involve the Sig-1R (Zanos et al., 2018). Given that the pKa of ketamine is 7.5 (Lester et al., 2015), ketamine will exist in almost equal concentration at its base form and protonated form according to Eq. 1 above. As an extension, the ketamine concentration inside a neuron will be about half of that in the blood. The standard ketamine infusion treatment for depression is 40 mg/40 ml i.v. infusion over 40 min (Wan et al., 2015). Therefore, the concentration of ketamine in the blood of a 75 kg person would be about 30 μM. The intracellular concentration of ketamine would therefore be about 15 μM, which would occupy about 10% of the Sig-1R given that the Ki of racemic ketamine at the Sig-1R is 139 μM (Robson et al., 2012). Of course, ketamine may also act on other intracellular targets such as the nascent NMDA receptor as indicated in a report (Lester et al., 2012).

Lastly, cannabinoid has recently been shown to exert its memory-impairment action through a non-canonical target, the cannabinoid type 1 receptor inside a neuron on the mitochondrial membrane (Hebert-Chatelain et al., 2016).

Thus, drugs of abuse, regardless of whether they are weak base or strongly lipophilic, may target, in addition to traditionally recognized receptors or transporters, non-canonical molecules either on the plasma membrane or inside a neuron to exert their action.

In summary, this perspective would call attention to looking into the non-canonical target of drugs of abuse which may play a key role in furthering our understanding of the action of drugs of abuse.

## Data Availability

All datasets generated for this study are included in the manuscript and/or the supplementary files.

## Ethics Statement

This study was carried out in accordance with the principles as indicated by the NIH Guide for the Care and Use of Laboratory Animals to assist in caring for and using animals in ways judged to be scientifically, technically, and humanely appropriate. The animal study was reviewed and approved by Dr. Alex Hoffman, IRP, NIDA, and NIH.

## Author Contributions

The author confirms being the sole contributor of this work and has approved it for publication.

## Conflict of Interest Statement

The author declares that the research was conducted in the absence of any commercial or financial relationships that could be construed as a potential conflict of interest. The handling Editor declared a past collaboration with the author.
